# Awareness and knowledge regarding female genital schistosomiasis among European healthcare workers: a cross-sectional online survey

**DOI:** 10.1186/s12992-024-01095-z

**Published:** 2025-01-08

**Authors:** Valentina Marchese, Aaron Remkes, Irina Kislaya, Pia Rausche, André Brito, Jana Christina Hey, Tahinamandranto Rasamoelina, Rivo Andry Rakotoarivelo, Jürgen May, Daniela Fusco

**Affiliations:** 1 Research Group: Implementation Research, Bernhard Nocht Institute for Tropical Medicine, Hamburg, Germany; 2 German Center for Infection Research (DZIF), Hamburg-Borstel-Lübeck-Riems, Germany; 3 Centre d’Infectiologie Charles Mérieux (CICM), University of Antananarivo, Antananarivo, 101 Madagascar; 4 Department of Infectious Diseases, University of Fianarantsoa Andrainjato, Fianarantsoa, 301 Madagascar; 5 University Medical Center Hamburg-Eppendorf (UKE), Hamburg, Germany; 6 Department of Infectious Diseases Epidemiology, Bernhard-Nocht Institute for Tropical Medicine, Hamburg, Germany

**Keywords:** Female genital Schistosomiasis, Public Health, Health literacy, Europe, Migrant health, Women’s health

## Abstract

**Background:**

Adequate knowledge and awareness regarding diseases are essential for appropriate, high-quality healthcare. Female Genital Schistosomiasis (FGS) is a non-sexually transmitted gynaecological disease that is caused by the presence of *Schistosoma haematobium* eggs in the female genital tract and the resulting immune response that causes tissue damage. It is estimated to affect 56 million women, mostly in sub-Saharan Africa (SSA), where healthcare workers (HCWs) have limited awareness and knowledge of FGS. Most migrants in Europe are female, often from SSA and therefore at risk of FGS. This study investigated awareness and knowledge of FGS among European HCWs with the aim of informing strategies to improve the management of migrant health.

**Methods:**

We conducted a cross-sectional survey using a self-administered, closed, multilingual, anonymous online questionnaire between 1st June 2023 to 31st January 2024. Medical doctors (MDs) (*n* = 581) and nurses or midwives (NMs) (*n* = 341) working in infectiology, gynaecology, urology and general, travel, internal or occupational medicine in European countries were enrolled in the survey. A Poisson regression was used to identify factors associated with MDs’ knowledge and awareness of FGS and adjusted prevalence ratios (aPR) were estimated. Practices related to FGS were described using counts and proportions for a subsample of MDs aware of FGS.

**Results:**

Among the 922 eligible participants, FGS awareness was 43.7% (CI95%: 39.6; 47.9) for MDs and 12.0% (CI95%: 8.8; 16.0) for NMs. FGS awareness among MDs was higher among men (50.0%; CI95%: 43.7; 56.3), working in clinics for migrants (72.0%, CI95%: 63.2; 79.7) and among infectiologists/travel medicine specialists (68.9%, CI95%: 62.2; 75.0). No knowledge was reported by 67.6% (95% CI 63.7–71.4) of MDs, while 25.3% (CI95%: 21.8; 29.0) had low and 7.1% (CI95%: 5.1; 9.5) medium knowledge. Working in healthcare for migrants was positively associated with medium knowledge (aPR = 3.49; CI95% 1.67;7.28), which was lower for general practitioners (aPR = 0.23, CI95%:0.07;0.81).

**Conclusions:**

Our study highlights that HCWs in Europe might not be adequately prepared to manage FGS patients, resulting in a high risk of neglect. We believe that the promotion of existing medical networks could improve knowledge about FGS and thus the health of migrant women.

**Supplementary Information:**

The online version contains supplementary material available at 10.1186/s12992-024-01095-z.

## Background

Healthcare workers’ (HCWs) knowledge and awareness of diseases contribute to all aspects of healthcare, particularly adequate diagnosis and clinical management [1, 2]. Their deficiency drives the emergence of unmet healthcare needs (UHCNs) [3], defined as the lack of services deemed necessary to prevent adverse health outcomes [4], particularly for Neglected Tropical Diseases (NTDs), which already receive low levels of attention worldwide. It has been shown that knowledge and awareness of these diseases are limited in several countries endemic to schistosomiasis and are still greatly unexplored in non-endemic settings. Lack of knowledge and awareness delays much needed diagnosis and treatment while fuelling the development of chronic and complicated manifestations, as in the case of schistosomiasis [5–7].

Female Genital Schistosomiasis (FGS) is a gynaecological disease caused by the deposition and calcification of schistosome eggs in the genital tract following continuous and/or persistent infection with *Schistosoma haematobium* [8]. The clinical symptoms are unspecific and resemble those of sexually transmitted infections (STIs), including vaginal bleeding, itching, discharge, and/or dyspareunia [8]. Possible complications of the disease include infertility, miscarriages, ectopic pregnancies and an increased risk of contracting STIs [9]. While the global burden of the disease, mostly due to under- or misdiagnosis, is not known, the condition is estimated to affect 56 million women and girls worldwide [10].

The diagnosis of FGS is complex as the recommended diagnostic tool is colposcopy, which is difficult to implement in resource-limited settings [11, 12] with most significant disease burden. Conventional methods used for the diagnosis of urinary schistosomiasis [13] can have detection limits for FGS as a specific biomarker for the disease is yet to be identified [14]. The lack of knowledge and awareness regarding FGS among HCWs reported in endemic countries exacerbates the diagnostic complexity, hinders accurate diagnosis and increases the risk of misclassification of FGS as an STIs [5, 15].

Recently, there has been growing evidence of the presence of schistosomiasis in Europe, mostly among migrant populations from endemic areas [16]. Currently, international and national guidelines for the medical care of these risk groups focus mainly on screening for this disease [17]. However, these guidelines generally miss recommendations for the management of complicated and chronic forms of the disease, such as FGS. Additionally, they are addressed within the scope of preventive medicine, tropical disease or health services provided for migrant and traveller populations [17, 18] whilst failing to reach other medical services such as gynaecology amongst others.

The management of chronic and non-communicable diseases among migrants is typically associated with various barriers linked to social, cultural and legal factors, which often prevent access to appropriate healthcare services required for multidisciplinary and adequate disease management and treatment [19].

Females represent more than 50% of the migrant population in Europe [20]. A recent survey of migrant women coming to Europe highlights that 40% are originating from sub-Saharan Africa (SSA) [21]. Evidence underlines that, migrant women in Europe face significant challenges to access healthcare, leading to a reduced utilisation of gynaecological services or STI screening, among others [22, 23].

Our leading hypothesis is that FGS in Europe may represent a hidden problem among migrant populations, and that the potential lack of knowledge and awareness of FGS among HCWs determines UHCNs for a considerable proportion of women from endemic countries who are at risk of the disease.

The objective of this study was to assess the knowledge and awareness of FGS among European HCWs with the overall aim to identify possible barriers to appropriate management of the disease in the region.

## Methods

### Study design and population

This study employed a cross-sectional design based on a self-administered, closed, online questionnaire assessing the level of awareness, knowledge, and practices regarding FGS among HCWs in Europe.

Inclusion criteria were: (i) being a HCW (doctor, nurse, or midwife), (ii) working in the field of infectiology, gynaecology, urology, general, travel, internal, family, and/or occupational medicine, (iii) working in a European country, (iv) aged ≥ 18 years, and (v) providing informed consent. No individual who met the inclusion criteria was excluded as a participant in this study based on sexual orientation, gender identity, political and ethnic affiliation, or socioeconomic position.

### Sample size and participant recruitment strategy

The minimum required sample size for the study was 1152 individuals (576 per professional category) in order to estimate independently for each professional category an expected prevalence of FGS awareness of 50% with margin of error of ± 5% at 95% confidence level assuming a design effect of 1.5. Samples size calculations were performed using OpenEpi software [24, 25]. Participants were contacted by email or telephone through professional networks, societies, private contacts, and social media groups. A convenience sampling methodology based on a snowball strategy was employed, promoting the survey by asking all participants to further disseminate the questionnaire within their networks. Flyers were distributed within relevant medical societies and conferences.

### Data collection and study instruments

Data were collected between the 1st of June 2023 and the 31st of January 2024. A link or QR code was disseminated to access the online questionnaire. This ensured anonymous data entry via an open-source survey tool on REDCap^®^ [26] hosted on a secured server at the Bernhard Nocht Institute for Tropical Medicine (Hamburg, Germany). Each participant was assigned a participant identifier (PID) to guarantee complete anonymisation. No personal or contact details were entered into any database. The survey was made available in German, Italian, English, French, Portuguese, and Spanish.

The questionnaire was structured to collect socio-demographic information with details on professional background, medical specialisation, and training, alongside awareness of schistosomiasis and FGS. Respondents indicating awareness of FGS were asked in an open-end question format to clarify their FGS-related training and comprehension of potential symptoms, complications, diagnostic modalities, screening tools, treatment options and their practices. In addition, participants were asked about their referral practices of FGS patients to other medical specialities. Open-ended responses were spell-checked, corrected, and categorised logically. Prior to launching the survey, the questionnaire was tested with at least one health professional speaking one of the six languages in which the survey would be administered. It was also reviewed by a total of two nurses, four clinicians, two psychologists and one epidemiologist.

### Statistical analysis

Descriptive statistics (counts and proportions) were used to characterise the study participants. The level of awareness of schistosomiasis and FGS, knowledge, and practices regarding FGS among HCWs were described through proportions and 95% confidence intervals (CI) stratified by professional category. A score was computed to evaluate the level of knowledge, with one point assigned for each correctly stated sign, symptom, complication, or diagnostic tool for FGS, as described in the World Health Organization (WHO) atlas [11, 27]. The total score (between 0 and 18 points) was classified as no (0 points), low (1–5 points), moderate (6–11 points), and high knowledge (12–18 points). Due to missing values, the denominators may vary in some analyses as only participants with complete information were included.

To identify factors associated with knowledge and awareness of FGS among medical doctors (MDs), crude and adjusted prevalence ratios (aPR) were estimated with 95% CIs using a Poisson regression with robust standard errors (SE). Regression modelling was not performed for the other professional categories due to the low proportion of nurses/midwives (NMs) being aware of FGS. Practices related to FGS were described using counts and proportions for a subsample of MDs aware of FGS. Data management and analysis were performed using Stata 15 and R v.3.2.1 [28, 29].

### Ethical considerations

The study was implemented and conducted according to the latest ICH Good Clinical Practice Guideline [30] and received a waiver by the Ethics Committee of the Hamburg State Medical Chamber (2023-300319-WF). Participants retained the right to refuse or withdraw from the study at any time without explanation. No financial incentives were provided for participation.

## Results

### Participants’ characteristics

Of a total of 1375 participants, 922 were considered eligible and included in the study (Figure S1). Of these, 63.0% (*n* = 581) were MDs and 37.0% (*n* = 341) were NMs. Participants’ characteristics are listed in Table 1. Overall, 69.6% of participants were women, although the distribution of respondents by gender varied considerably between MDs and NMs (55.1% vs. 94.4%). The distribution of participants across age groups varied from 20.8% for those 55 + years old to 29.0% for those aged < 34 in the overall sample.

**Table 1:** Participants’ characteristics

	Doctors		Nurses/Midwives		Overall sample	
	n	%	n	%	n	%
Gender						
Men	258	44.9	19	5.6	277	30.2
Women	317	55.1	322	94.4	639	69.8
Age group						
< 34 years old	176	30.3	91	26.7	267	29.0
35–44	150	25.8	108	31.7	258	28.0
45–54	117	20.1	88	25.8	205	22.2
55 + years old	138	23.8	54	15.8	192	20.8
Country of practice						
Germany	176	30.3	78	22.9	254	27.6
Italy	139	23.9	31	9.1	170	18.4
UK, Portugal, France*	100	17.2	168	49.3	268	29.1
Other	166	28.6	64	18.8	230	25.0
Years of experience						
0 to 9	203	36.8	95	28.7	298	33.8
10 to 29	239	43.3	173	52.3	412	46.7
30+	110	19.9	63	19.0	173	19.6
Highest degree						
High school diploma	na	na	39	11.6	39	4.4
Vocational training	na	na	50	14.9	50	5.6
Bachelor/undergraduate	na	na	75	22.3	75	8.4
Master/postgraduate	175	31.5	161	47.9	336	37.7
Specialised doctor/PhD	381	68.5	11	3.3	392	44.0
Work in the field of STIs						
No	516	88.8	331	97.1	847	91.9
Yes	65	11.2	10	2.9	75	8.1
Work with travellers/migrant populations						
No	456	78.5	322	94.4	809	84.4
Yes	125	21.5	19	5.6	113	15.6
Workplace						
Research/University hospital	249	43.2	46	13.6	295	32.2
Non-university hospital	132	22.9	45	13.3	177	19.3
Outpatient/medical practice	176	30.5	227	67.0	403	44.0
Other	20	3.5	21	6.2	41	4.5
Specialization						
None	28	4.8	na	na	na	na
Occupational medicine	30	5.2	na	na	na	na
Urology	31	5.3	na	na	na	na
Gynaecology	91	15.7	na	na	na	na
Internal medicine	67	11.5	na	na	na	na
Infectiology/travel/tropical	212	36.5	na	na	na	na
Family/General medicine	122	21.0	na	na	na	na
Specialization						
Gynaecology/maternal health	na	na	240	70.4	na	na
Other	na	na	101	29.6	na	na

Note: na = not applicable, *Countries were grouped per presence and arrival of migrant people and of people coming from Sub-Saharan countries. Italy had the highest number of arrivals in 2022, and it is among the countries with the highest presence of sub-Saharans; Germany registered the highest number of presence in 2022; Portugal, UK and France are among the countries with the highest presence of sub-Saharan people (29.31)

Of all MDs, 30.3% were practicing in Germany, 23.9% in Italy, 17.2% in the UK, Portugal, or France, and 28.6% in other European countries. Among NMs, 49.3% were working in the UK, Portugal, or France and only 9.1% in Italy. Most MDs (68.5%) were specialised or reported to have obtained a PhD degree: 36.5% were specialised in infectiology/travel/tropical medicine, 21.0% in general or family medicine, and 15.7% in gynaecology. Among NMs, 37.7% had a master’s degree and 44.0% held a PhD. Most NMs were working in the field of gynaecology/maternal health (70.4%).

Participants from both professional categories had similar years of working experience with an overall 33.8% reporting to have less than ten years, while 19.6% had more than 30 years of experience. MDs most frequently reported university hospitals or public research institutions as their main workplace (43.2%), while among NMs the most represented group was working in outpatient services or private medical practices (67.0%). The proportion of those who were working with travellers and migrant populations (21.5% vs. 5.6%) or in the area of STIs (11.2% vs. 2.9%) was higher among MDs compared to NMs.

### Awareness of schistosomiasis and FGS

**Fig. 1:**
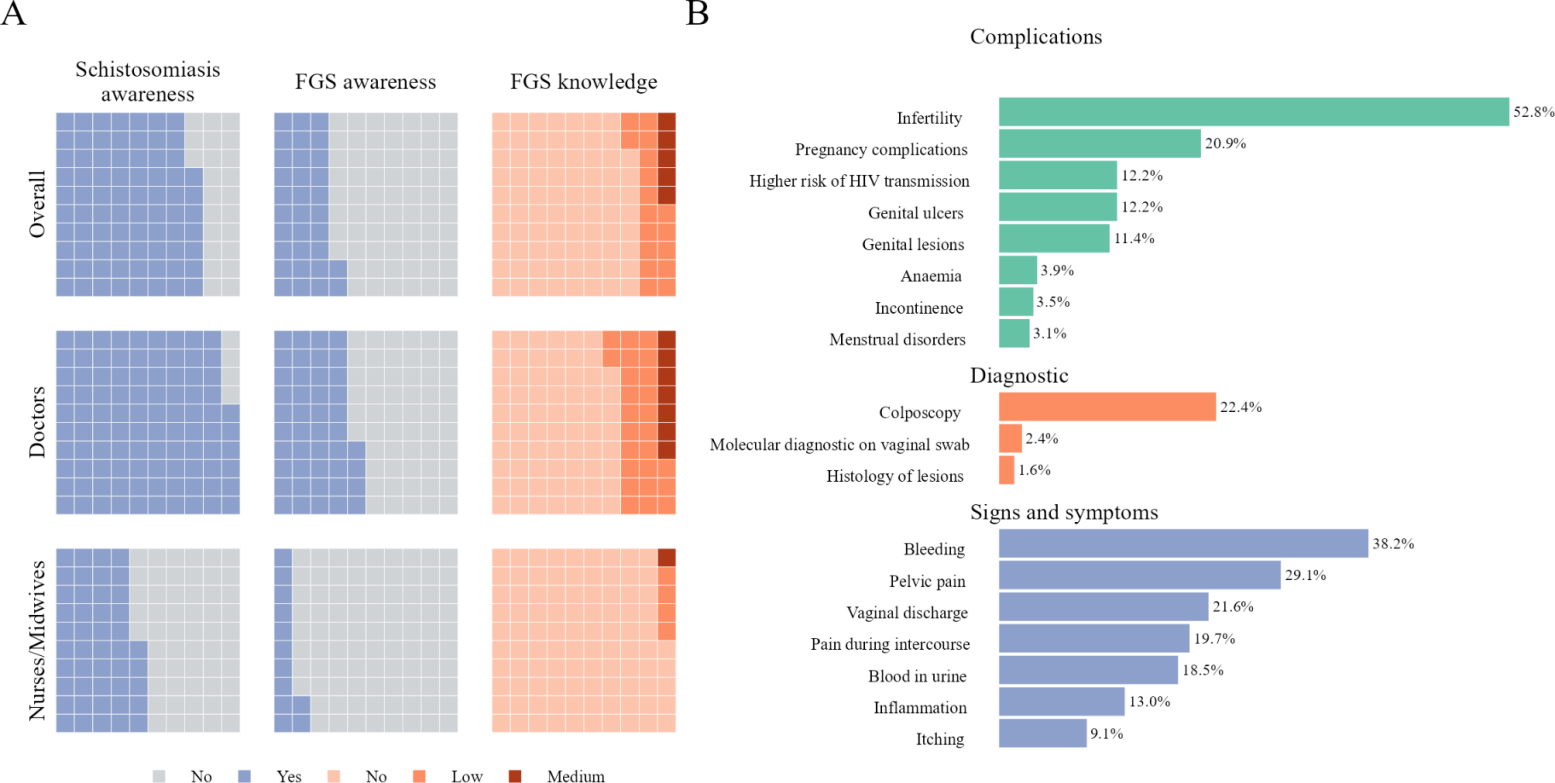
**(A)** Estimates of the prevalence of awareness of schistosomiasis, FGS awareness among medical doctors, nurses or midwives in European countries. **(B)** Diagnostic tools, signs, symptoms, and complications of FGS recognized by medical doctors aware of FGS in European countries

A total of 712 (77.2%, CI74.4; 79.8) participants reported to have heard about schistosomiasis before the survey. The level of awareness of schistosomiasis was 96.2% (CI: 94.3; 97.6) among MDs and 44.8% (CI: 39.5; 50.3) among NMs (Fig. 1). The level of FGS awareness was 43.7% (CI: 39.6; 47.9) for MDs, and lower at 12.0% (CI: 8.8; 16.0) for NMs, resulting in an overall low FGS awareness of 32.0% (CI: 29.1; 35.1) among all HCWs.

**Fig. 2:**
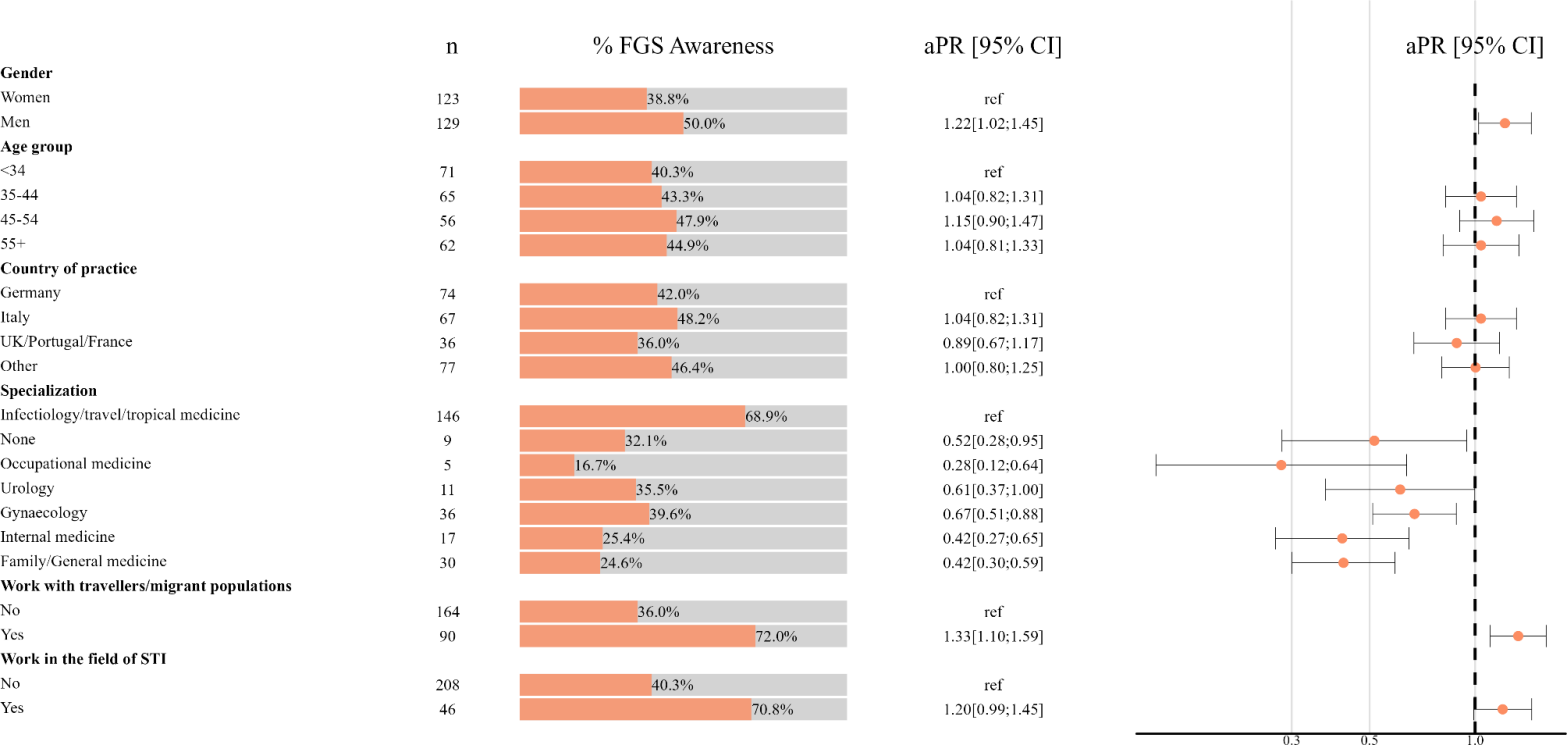
Estimates of the prevalence of FGS awareness and adjusted prevalence ratios of FGS awareness among medical doctors in European countries

For MDs, we observed a higher level of FGS awareness among men (50.0%; CI: 43.7; 56.3) compared to women (38.8%, CI: 33.4; 44.4) (Fig. 2 and Supplementary Table 1). MDs working in the field of STIs or with migrant populations/travellers showed the highest levels of FGS awareness at 70.8% (CI: 58.2; 81.4) and 72.0% (CI: 63.2; 79.7), respectively. FGS awareness varied between medical specialties, ranging from 16.7% (CI: 5.6; 34.7) for occupational medicine to 68.9% (CI 62.2; 75.0) for infectiology/travel/tropical medicine.

Among those aware of FGS, the academic curriculum (34.6% MDs and 36.6% NMs) followed by scientific literature (28.4% MDs, 17.1% NMs), were mentioned most frequently by both professional categories as source of information about FGS (Supplementary Table 2).

### Factors associated with medical doctors’ awareness of FGS

Adjusted prevalence ratios (aPR) show the increased prevalence of FGS awareness for men (aPR = 1.22 CI: 1.02; 1.45), and those working with migrant populations/travellers (aPR = 1.33, CI: 1.10; 1.59). In contrast, compared to infectiology/travel/tropical medicine, the awareness of FGS was lower among occupational medicine (aPR = 0.28; CI: 0.12; 0.64), gynaecology (aPR = 0.67; CI 0.51; 0.88), internal medicine (aPR = 0.42, CI: 0.27; 0.65), family/general medicine (aPR = 0.42, CI: 0.30; 0.59), and those without specialisation (aPR = 0.52, CI: 0.28; 0.95). No association was observed among the prevalence of FGS awareness and age groups, country of practice and working in the field of STIs.

### FGS knowledge

**Fig. 3:**
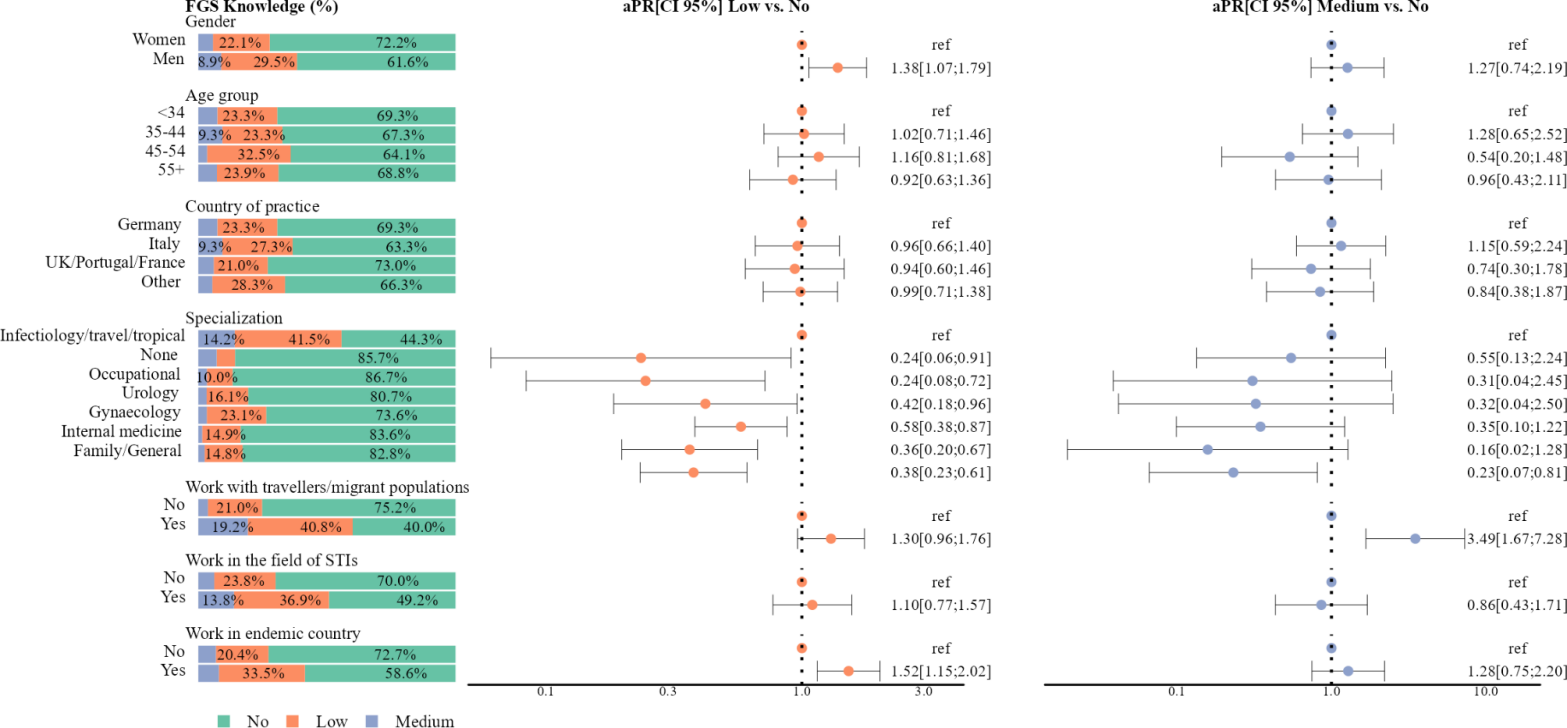
Levels of FGS knowledge and adjusted prevalence ratios of low and medium FGS knowledge among medical doctors in European countries

Among MDs, only 7.1% (CI: 5.1; 9.5) indicated medium knowledge of FGS, 25.3% (CI: 21.8; 29.0) had low knowledge, and 67.6% (CI: 63.7; 71.4) had no knowledge (Fig. 1). Among NMs the vast majority of respondents had no knowledge of FGS 95.3% (CI: 92.5; 97.3), while 4.4% (CI: 2.5; 7.2) and 0.3% (CI: 0.0; 1.6) had low and medium knowledge, respectively. None of the participants included in this study reached a high knowledge score regarding FGS (Fig. 3 and Supplementary Table 3). Most frequently, MDs being aware of FGS mentioned bleeding (38.2%) and pelvic pain (29.1%) as symptoms of the disease, infertility (52.8%) as its complication, and 22.4% identified colposcopy as a possible tool for FGS diagnosis (Fig. 1).

### Factors associated with knowledge of FGS among medical doctors

Factors associated with knowledge of FGS were assessed for both low and medium levels of knowledge, compared to no knowledge. The adjusted prevalence ratios (aPR) showed positive associations between low knowledge for men (aPR = 1.38, CI: 1.07; 1.79) and having worked in endemic countries (aPR = 1.52, CI: 1.15; 2.02) (Fig. 3 and Supplementary Table 4).

Additionally, compared to infectiology/travel/tropical medicine, all other specialties, namely, occupational medicine (aPR = 0.24; CI: 0.08; 0.72), urology (aPR = 0.42; CI: 0.18; 0.96), gynaecology (aPR = 0.58; CI: 0.38; 0.87), internal medicine (aPR = 0.36, CI: 0.20; 0.67), family/general medicine (aPR = 0.38, CI: 0.23; 0.61), and those without specialisation (aPR = 0.24, CI: 0.06; 0.91) were less likely to report low FGS knowledge.

Medium FGS knowledge was associated with working with travellers/migrant populations (aPR = 3.49; CI: 1.67; 7.28). In contrast, reporting medium knowledge of FGS was 77% lower (aPR = 0.23, CI: 0.07; 0.81) among MDs in the field of family/general medicine compared to infectiology/travel/tropical medicine specialists (Fig. 3).

### FGS practices

**Fig. 4:**
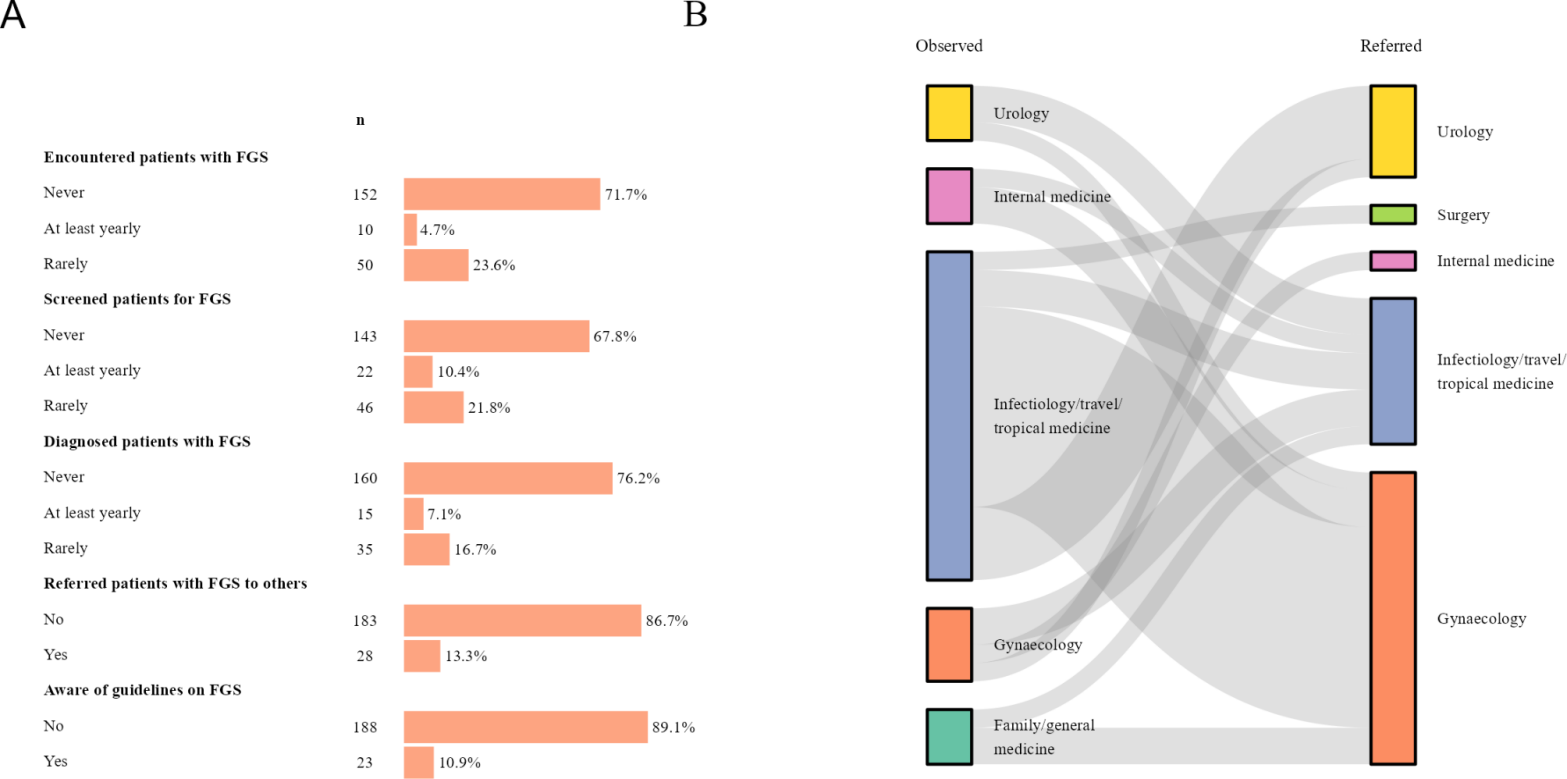
**A.** FGS practices in European countries. **B**. Referral pathway for FGS-suspected or diagnosed patients

Most MDs aware of FGS reported little practical experience with the disease, with 71.7% having never encountered FGS patients and 76.2% having never diagnosed the condition. Only 10.4% and 7.1% of MDs reported that they screened patients for FGS or diagnosed FGS annually, respectively (Fig. 4, Panel A). A total of 28 MDs indicated referring FGS cases to HCWs of other specialities. Referrals to gynaecology and infectiology/tropical medicine were most frequently mentioned (Fig. 4, Panel B). A small proportion of participants stated being aware of national and international guidelines on schistosomiasis and/or FGS. WHO and national guidelines were mainly mentioned.

## Discussion

This cross-sectional survey reports an overall low awareness 32.0% (CI: 29.1; 35.1) and poor knowledge (7.1% medium knowledge and 25.3% low knowledge) of FGS among HCWs, raising concerns about the capacity to identify and recognise the disease in at-risk patients in Europe. Although several studies have explored knowledge and awareness of FGS in endemic countries [5, 15, 31, 32], to the best of our knowledge, this marks the first of its kind in Europe. Even though the established endemicity of schistosomiasis in southern Europe [33] does not appear to lead to a high disease burden in Europe, schistosomiasis and especially its chronic forms could go unnoticed despite being prevalent in marginalised groups such as migrant populations [34–36].

The 2030 Agenda for Sustainable Development acknowledges migration as a critical driver for global sustainable development, benefiting both migrant and host communities [37]. More than one of the Sustainable Development Goals (SDGs) recognises the multidimensional aspect of migration, which requires a multisectoral coordination “to leave no one behind”, especially with regards to health [38]. Universal Health Coverage (UHC) and the availability and provision of the highest standard of health for all are key to achieving health and well-being of migrant and displaced people [39].

Both the European Union and the WHO European Region have developed strategies and guidelines to improve the health of migrant people [40–42]. However, in the past, migrant health has been associated with the control of communicable and outbreak-prone diseases [43], with the primary objective being to safeguard host communities [44]. Accordingly, less attention has been dedicated to chronic diseases [19], although various strategies have been proposed for their adequate management [17, 45]. Limited access to health services due to legal, structural or individual barriers further exacerbates this vicious cycle, limiting the successful management of migrants’ health [19]. This is further complicated by the lack of knowledge regarding certain diseases that are typical of endemic countries and are infrequent in Europe, such as schistosomiasis and its chronic consequences, across all levels of care. Furthermore, there is still no consensus on the screening and diagnosis of schistosomiasis in non-endemic areas, especially with regard to the identification of chronic manifestations. Serology is currently the recommended method for the screening of schistosomiasis in migrant populations [17]. Nevertheless, this method has limitations in terms of specificity and sensitivity, which add to the non-specificity of the clinical manifestations of chronic schistosomiasis and the need for second-level diagnostic methods, such as colposcopy, to confirm chronic disease, which are themselves often still under debate. Our study reveals that among HCWs who are relatively well aware of the existence of schistosomiasis (77.2%, CI: 74.4; 79.8), as also previously shown by Mantica et al. [46], awareness and, more concerningly, knowledge of FGS are notably lacking. Not surprisingly, our data show that MDs working in infectiology/travel medicine (68.9%, CI: 62.2; 75.0) and clinicians with more direct contact with migrant people (72.0%, CI: 63.2; 79.7) have a higher level of awareness of FGS. The finding that men (50.0%, CI: 43.7; 56.3) have a higher awareness of FGS is interesting, especially in light of recent suggestions of better clinical outcomes for female patients in case of gender concordance with the treating HCWs [47] and the facilitating role of the presence of women HCWs in the provision of health care to migrant women [48]. However, this difference was not confirmed for mid-level FGS knowledge. The implications should be further explored. Noteworthy is that specialisations such as family medicine (24.6%, CI: 17.3; 33.2) and occupational medicine (16.7%, CI: 5.6; 34.7) are the categories with lower knowledge of the disease, although these are the healthcare specialities that are more likely to be the first point of access to the healthcare system for women experiencing consequences of FGS. The data on the poor awareness and knowledge of FGS among NMs is concerning and warrants further investigation due to its potential public health implications. NMs are often at the forefront of community-based interventions and screening, which is currently the main method for addressing schistosomiasis in migrant populations. A lack of awareness and knowledge could have a major impact on screening procedures, especially given the diagnostic limitations mentioned earlier. If FGS is not suspected during screening, it is highly likely that patient will experience a cycle of misdiagnosis and mistreatment, as already observed in endemic countries. This situation is further complicated by barriers to healthcare access linked to their migrant status. The consequences include the risk of chronic suffering and an increased vulnerability to acquiring HIV, which in Europe has a high rate of post-migration transmission [49]. Most encouragingly, of the respondents who had ever referred a patient with a suspected or diagnosed FGS to another doctor (*n* = 28), the majority chose a gynaecologist (53.6%). This finding is limited to a very small number of participants, hence no conclusions can be drawn, although it does suggest that the referral pathway for effective management of FGS seems to be clear.

Our data show that most of the knowledge about FGS among MDs is acquired through academic curricula (34.7%), scientific literature (28.4%) and conferences (25.6%). This suggests that academia, including specialty-specific curricula, still plays a crucial role in the development of knowledge in HCWs and should be expanded to include FGS. Interestingly, it has been shown that to be up to date, MDs should spend an average of four hours per week reading scientific publications, but alarmingly just few specialists report to dedicate this time to reading [50]. Continuing medical education (CME) is often accomplished by attending conferences [51, 52]. The exponential increase in events sponsored by the pharmaceutical sector [51] is detrimental towards NTDs, whose specific treatments are neither officially licensed nor marketed in Europe [53, 54] and have consequently lower representation at such events. These data are aligned with the challenges of management and detection of both FGS and other complicated forms of schistosomiasis reported in Europe [35, 55, 56], and demonstrate the urgency of restructuring CME to ensure that HCWs have adequate access to relevant medical knowledge oriented towards the needs of patients and the changing demands created by the dynamic societies that shape our globe. The development of multidisciplinary and translational CME events that include basic migrant health concepts for HCWs of different specialties would help to overcome the under-representation of FGS and other NTDs, and overall promote migrant-sensitive healthcare at different levels, both biomedical and service-oriented. The effort to find solutions for more effective management of neglected conditions such as FGS in the European region is evident and is underscored by the existence of networks and scientific societies such as TropNet and FESTMIH [57, 58], which are engaged in establishing transnational registries among migrant populations and contributing to the reform of CME.

Despite its uniqueness and strengths of the results presented, this study does not come without limitations. The biggest limitation of the study was the adoption of a convenience sampling strategy, that might have produced some selection bias. Namely, men NMs and MDs older than 55 years old might be underrepresented in our sample, affecting the generalisability of the study findings. In addition, due to use of a snowball sampling strategy, we were not able to compute a survey participation rate or to collect any data on survey non-participants. Thus, we cannot completely exclude that those with lower awareness of FGS did not reply to the survey, leading to an overestimation of FGS awareness prevalence. Finally, low number of recruited NMs affected the precision of the estimates for this professional category, and limited our ability to conduct association analysis among NMs.

In conclusion, our study highlights that European HCWs may not be adequately prepared to deal with diseases that are gaining relevance on the European continent due to the global connectivity and the dynamic nature of our societies. Our data suggests that FGS is at great risk of being neglected by the European healthcare system, limiting the achievement of UHC by 2030 as set out in the SDGs. We believe that the commitment of European countries to address these shortcomings is clear, though important gaps remain to be filled to reach the last mile.

## Electronic supplementary material

Below is the link to the electronic supplementary material.

Supplementary Material 1

Supplementary Material 2

## Data Availability

No datasets were generated or analysed during the current study.

## References

[1] Alshdaifat E, Sindiani A, Amarin Z, et al. Awareness of polycystic ovary syndrome: a university students’ perspective. Annals Med Surg. 2021;72:103123. 10.1016/j.amsu.2021.103123 PMC8654774 34934483

[2] Sinan I, Mihdawi M, Farahat AR, Fida M. Knowledge and awareness of Rare diseases among Healthcare professionals in the Kingdom of Bahrain. Cureus 15: e47676. 10.7759/cureus.47676 PMC10673629 38022232

[3] Kpessa-Whyte M. Aging and demographic transition in Ghana: state of the Elderly and Emerging issues. Gerontologist. 2018;58:403–8. 29381779 10.1093/geront/gnx205

[4] Vreman RA, Heikkinen I, Schuurman A, et al. Unmet Medical need: an introduction to definitions and stakeholder perceptions. Value Health. 2019;22:1275–82. 31708064 10.1016/j.jval.2019.07.007

[5] Rausche P, Rakotoarivelo RA, Rakotozandrindrainy R, et al. Awareness and knowledge of female genital schistosomiasis in a population with high endemicity: a cross-sectional study in Madagascar. Front Microbiol. 2023;14:1278974. 37886060 10.3389/fmicb.2023.1278974 PMC10598593

[6] Chami GF, Bundy DAP. More medicines alone cannot ensure the treatment of neglected tropical diseases. Lancet Infect Dis. 2019;19:e330–6. 31160190 10.1016/S1473-3099(19)30160-4

[7] Elfar E, Asem N, Yousof H. The awareness of neglected tropical diseases in a sample of medical and nursing students in Cairo University, Egypt: a cross-sectional study. PLoS Negl Trop Dis. 2020;14:e0008826. 33206641 10.1371/journal.pntd.0008826 PMC7673504

[8] Kjetland EF, Leutscher PDC, Ndhlovu PD. A review of female genital schistosomiasis. Trends Parasitol. 2012;28:58–65. 22245065 10.1016/j.pt.2011.10.008

[9] World Health Organization. Female genital schistosomiasis: simultaneous screening of diseases can improve reproductive health. WHO News 2018. https://www.who.int/news/item/20-07-2018-female-genital-schistosomiasis-simultaneous-screening-of-diseases-can-improve-reproductive-health (accessed 23 Apr 2024).

[10] World Health Organization & UNAIDS. No more neglect — female genital schistosomiasis and HIV — integrating sexual and reproductive health interventions to improve women’s lives. Geneva, 2019.

[11] World Health Organization. Female genital schistosomiasis. A pocket atlas for clinical health-care professionals. Genève: Organisation mondiale de la Santé. 2018. https://www.unaids.org/sites/default/files/media_asset/female_genital_schistosomiasis_and_hiv_en.pdf (accessed 23 Apr 2024).

[12] Xue P, Ng MTA, Qiao Y. The challenges of colposcopy for cervical cancer screening in LMICs and solutions by artificial intelligence. BMC Med. 2020;18:169. 32493320 10.1186/s12916-020-01613-x PMC7271416

[13] World Health Organization. Schistosomiasis. Fact sheets 2023. https://www.who.int/news-room/fact-sheets/detail/schistosomiasis (accessed 10 Nov 2024).

[14] Bustinduy AL, Randriansolo B, Sturt AS, et al. An update on female and male genital schistosomiasis and a call to integrate efforts to escalate diagnosis, treatment and awareness in endemic and non-endemic settings: the time is now. Advances in Parasitology. Elsevier,; 2022. 10.1016/bs.apar.2021.12.003 35249661

[15] Mazigo HD, Samson A, Lambert VJ, et al. Female genital schistosomiasis is a sexually transmitted disease: gaps in healthcare workers’ knowledge about female genital schistosomiasis in Tanzania. PLOS Glob Public Health. 2022;2:e0000059. 36962298 10.1371/journal.pgph.0000059 PMC10021524

[16] Asundi A, Beliavsky A, Liu XJ, et al. Prevalence of strongyloidiasis and schistosomiasis among migrants: a systematic review and meta-analysis. Lancet Global Health. 2019;7:e236–48. 30683241 10.1016/S2214-109X(18)30490-X

[17] European Centre for Disease Prevention and Control. Public health guidance on screening and vaccination for infectious diseases in newly arrived migrants within the EU/EEA. LU: Publications Office. 2018. https://data.europa.eu/doi/10.2900/154411 (accessed 22 Apr 2024).

[18] Comelli A, Genovese C, Gobbi F, et al. Schistosomiasis in non-endemic areas: Italian consensus recommendations for screening, diagnosis and management by the Italian Society of Tropical Medicine and Global Health (SIMET), endorsed by the Committee for the Study of Parasitology of the Italian Association of Clinical Microbiologists (CoSP-AMCLI), the Italian Society of Parasitology (SoIPa), the Italian Society of Gastroenterology and Digestive Endoscopy (SIGE), the Italian Society of Gynaecology and Obstetrics (SIGO), the Italian Society of Colposcopy and Cervico-Vaginal Pathology (SICPCV), the Italian Society of General Medicine and Primary Care (SIMG), the Italian Society of Infectious and Tropical Diseases (SIMIT), the Italian society of pediatrics (SIP), the Italian Society of Paediatric Infectious Diseases (SITIP), the Italian Society of Urology (SIU). Infection. 2023;51:1249–71. 37420083 10.1007/s15010-023-02050-7 PMC10545632

[19] World Health Organization. Continuum of Care for Noncommunicable Disease Management During the Migration Cycle. (1st ed.). Geneva: World Health Organization. 2022. https://www.afro.who.int/sites/default/files/2023-04/9789240044401-eng.pdf (accessed 11 Oct 2024). 35536925

[20] International Office for Migration. Data interactive dashboard. Share of female migrants in Europe. Migration data portal 2021. https://www.migrationdataportal.org/dashboard/compare-geographic (accessed 10 Nov 2024).

[21] International Office for Migration. Mixed migration flows – women & migration. Women and girls on the move to Europe 2018–2020. Austria: Vienna; 2021.

[22] Keygnaert I, Guieu A, Ooms G, Vettenburg N, Temmerman M, Roelens K. Sexual and reproductive health of migrants: does the EU care? Health Policy. 2014;114:215–25. 24268324 10.1016/j.healthpol.2013.10.007

[23] World Health Organization. Women on the move: migration, care work and health. Geneva: World Health Organization; 2017. https://iris.who.int/handle/10665/259463. (accessed 10 Nov 2024).

[24] Sullivan KM, Dean A, Soe MM. OpenEpi: a web-based epidemiologic and statistical calculator for public health. Public Health Reports (Washington, D.C.: 1974) 2009. 10.1177/003335490912400320 PMC2663701 19445426

[25] Scheaffer RL, Mendenhall W, Ott L, Gerow K. Elementary survey sampling. Fourth Edition. CA, USA: Duxbury Press Belmont; 1990.

[26] Harris PA, Taylor R, Thielke R, Payne J, Gonzalez N, Conde JG. Research electronic data capture (REDCap)—A metadata-driven methodology and workflow process for providing translational research informatics support. J Biomed Inform. 2009;42:377–81. 18929686 10.1016/j.jbi.2008.08.010 PMC2700030

[27] World Health Organization. Increasing awareness on genital manifestations of schistosomiasis. 2018. https://www.who.int/activities/increasing-awareness-on-genital-manifestations-of-schistosomiasis (accessed 5 Jun 2024).

[28] Stata Corp. Stata Statistical Software: Release 15. 2023. https://www.stata.com/

[29] R Core Team. R: A Language and Environment for Statistical Computing. 2024. https://www.R-project.org

[30] International Council for Harmonisation of Technical Requirements for Pharmaceuticals for Human Use. ICH E6 (R3) Guideline on good clinical practice (GCP)_Step 2b. 2023. https://www.ema.europa.eu/en/ich-e6-r2-good-clinical-practice-scientific-guideline

[31] Aribodor OB, Mogaji HO, Surakat OA, et al. Profiling the knowledge of female medical/para-medical students, and expertise of health care professionals on female genital schistosomiasis in Anambra, South Eastern Nigeria. PLoS Negl Trop Dis. 2023;17:e0011132. 36795786 10.1371/journal.pntd.0011132 PMC9977039

[32] Yirenya-Tawiah DR, Ackumey MM, Bosompem KM. Knowledge and awareness of genital involvement and reproductive health consequences of urogenital schistosomiasis in endemic communities in Ghana: a cross-sectional study. Reprod Health. 2016;13:117. 27655032 10.1186/s12978-016-0238-5 PMC5031356

[33] Rothe C, Zimmer T, Schunk M, et al. Developing endemicity of Schistosomiasis, Corsica, France. Emerg Infect Dis. 2021;27:319–21. 33264582 10.3201/eid2701.204391 PMC7774576

[34] Beltrame A, Buonfrate D, Gobbi F, et al. The hidden epidemic of schistosomiasis in recent African immigrants and asylum seekers to Italy. Eur J Epidemiol. 2017;32:733–5. 28560535 10.1007/s10654-017-0259-6

[35] Zammarchi L, Gobbi F, Angheben A, et al. Schistosomiasis, strongyloidiasis and Chagas disease: the leading imported neglected tropical diseases in Italy. J Travel Med. 2020;27:taz100. 31840757 10.1093/jtm/taz100

[36] Roure S, Valerio L, Pérez-Quílez O, et al. Epidemiological, clinical, diagnostic and economic features of an immigrant population of chronic schistosomiasis sufferers with long-term residence in a non-endemic country (North Metropolitan area of Barcelona, 2002–2016). PLoS ONE. 2017;12:e0185245. 28953954 10.1371/journal.pone.0185245 PMC5617205

[37] United Nations General Assembly. Transforming our world: the 2030 Agenda for Sustainable Development. Resolution A/RES/70/1, 21 October. 2015. https://sdgs.un.org/2030agenda (accessed 23 Apr 2024).

[38] International Office for Migration. Global Migration Data Analysis Centre. Migration Data Portal. Migration data and the sustainable development goals (SDGs). Migration data portal 2023. https://www.migrationdataportal.org/sdgs (accessed 11 Apr 2024).

[39] World Health Organization. The World Health Assembly extends the global action plan for refugee and migrant health until 2030. 2023. https://www.who.int/news/item/26-05-2023-the-world-health-assembly-extends-the-global-action-plan-for-refugee-and-migrant-health-until-2030 (accessed 22 Apr 2024).

[40] World Health Organization. Action plan for refugee and migrant health in the WHO European Region 2023–2030. Copenhagen: WHO Regional Office for Europe; 2023. https://www.who.int/europe/publications/i/item/WHO-EURO-2023-8966-48738-72475. (accessed 28 Mar 2024).

[41] World Health Organization. Strategy and Action Plan for Refugee and migrant health in the WHO European Region 2016–2022. Copenhagen: WHO Regional Office for Europe; 2016. https://www.who.int/europe/publications/i/item/EUR-RC66-8. (accessed 28 Mar 2024).

[42] European Centre for Disease Prevention and Control. Expert opinion on the public health needs of irregular migrants, refugees or asylum seekers across the EU’s southern and south-eastern borders. LU: Publications Office; 2015. https://data.europa.eu/doi/10.2900/58156. accessed 28 Mar 2024).

[43] Dara M, Gushulak BD, Posey DL, Zellweger J-P, Migliori GB. The history and evolution of immigration medical screening for tuberculosis. Expert Rev Anti-infective Therapy. 2013. 10.1586/eri.12.168. 23409820 10.1586/eri.12.168

[44] European Centre for Disease Prevention and Control., Italian Institute of Public Health. Handbook on implementing syndromic surveillance in migrant reception/detention centres and other refugee settings: technical document. LU: Publications Office. 2016. https://data.europa.eu/doi/10.2900/22710 (accessed 28 Mar 2024).

[45] Lotfi T, Itani MI, Howeiss P, Kilzar L, Rizk NA, Akl EA. Practice guidelines on migrants’ health: assessment of their quality and reporting. Health Qual Life Outcomes. 2020;18:125. 32380997 10.1186/s12955-020-01363-7 PMC7204216

[46] Mantica G, Van der Merwe A, Terrone C, et al. Awareness of European practitioners toward uncommon tropical diseases: are we prepared to deal with mass migration? Results of an international survey. World J Urol. 2020;38:1773–86. 31538244 10.1007/s00345-019-02957-7

[47] Lau ES, Hayes SN, Volgman AS, et al. Does patient-physician gender concordance influence patient perceptions or outcomes? J Am Coll Cardiol. 2021;77:1135–8. 33632488 10.1016/j.jacc.2020.12.031

[48] Filler T, Jameel B, Gagliardi AR. Barriers and facilitators of patient centered care for immigrant and refugee women: a scoping review. BMC Public Health. 2020;20:1013. 32590963 10.1186/s12889-020-09159-6 PMC7318468

[49] Nöstlinger C, Cosaert T, Landeghem EV, et al. HIV among migrants in precarious circumstances in the EU and European Economic Area. Lancet HIV. 2022;9:e428–37. 35460600 10.1016/S2352-3018(22)00032-7

[50] Garba S, Ahmed A, Mai A, Makama G, Odigie V. Proliferations of Scientific Medical journals: a burden or a blessing. Oman Med J. 2010;25:311–4. 22043365 10.5001/omj.2010.89 PMC3191655

[51] Ahmed K, Wang TT, Ashrafian H, Layer GT, Darzi A, Athanasiou T. The effectiveness of continuing medical education for specialist recertification. Can Urol Assoc J. 2013;7:266–72. 24032064 10.5489/cuaj.378 PMC3758945

[52] Davis D, O’Brien MA, Freemantle N, Wolf FM, Mazmanian P, Taylor-Vaisey A. Impact of formal continuing medical education: do conferences, workshops, rounds, and other traditional continuing education activities change physician behavior or health care outcomes? *JAMA* 1999; 282: 867–74. 10.1001/jama.282.9.867 10478694

[53] Calleri G, Angheben A, Albonico M. Neglected tropical diseases in Europe: rare diseases and orphan drugs? Infection. 2019;47:3–5. 30390200 10.1007/s15010-018-1241-2

[54] Buonfrate D, Tamarozzi F, Gobbi AF. Imported chronic schistosomiasis: screening and management issues. J Travel Med. 2020;27:taaa005. 31967311 10.1093/jtm/taaa005

[55] Roure S, Vallès X, Pérez-Quílez O et al. Female genitourinary schistosomiasis-related symptoms in long-term sub-saharan African migrants in Europe: a prospective population-based study. J Travel Med 2024; taae035. 10.1093/jtm/taae035 PMC11298046 38438139

[56] Comelli A, Riccardi N, Canetti D, et al. Delay in schistosomiasis diagnosis and treatment: a multicenter cohort study in Italy. J Travel Med. 2020;27:taz075. 31616948 10.1093/jtm/taz075

[57] The European Network for Tropical Medicine and Travel Health. TropNet. http://tropnetdev.netsysco.net/ (accessed 11 Apr 2024).

[58] Federation of European societies for tropical medicine and international health. FESTMIH | Home. https://www.festmih.eu/ (accessed 11 Apr 2024). 10.1111/j.1365-3156.2005.01469.x 16045468

