# Supplementary figures and images for: Awareness and knowledge regarding female genital schistosomiasis among European healthcare workers: a cross-sectional online survey
Source: Global Health. 2025 Jan 8;21:2. doi: 10.1186/s12992-024-01095-z (PMC11715917; doi:10.1186/s12992-024-01095-z)

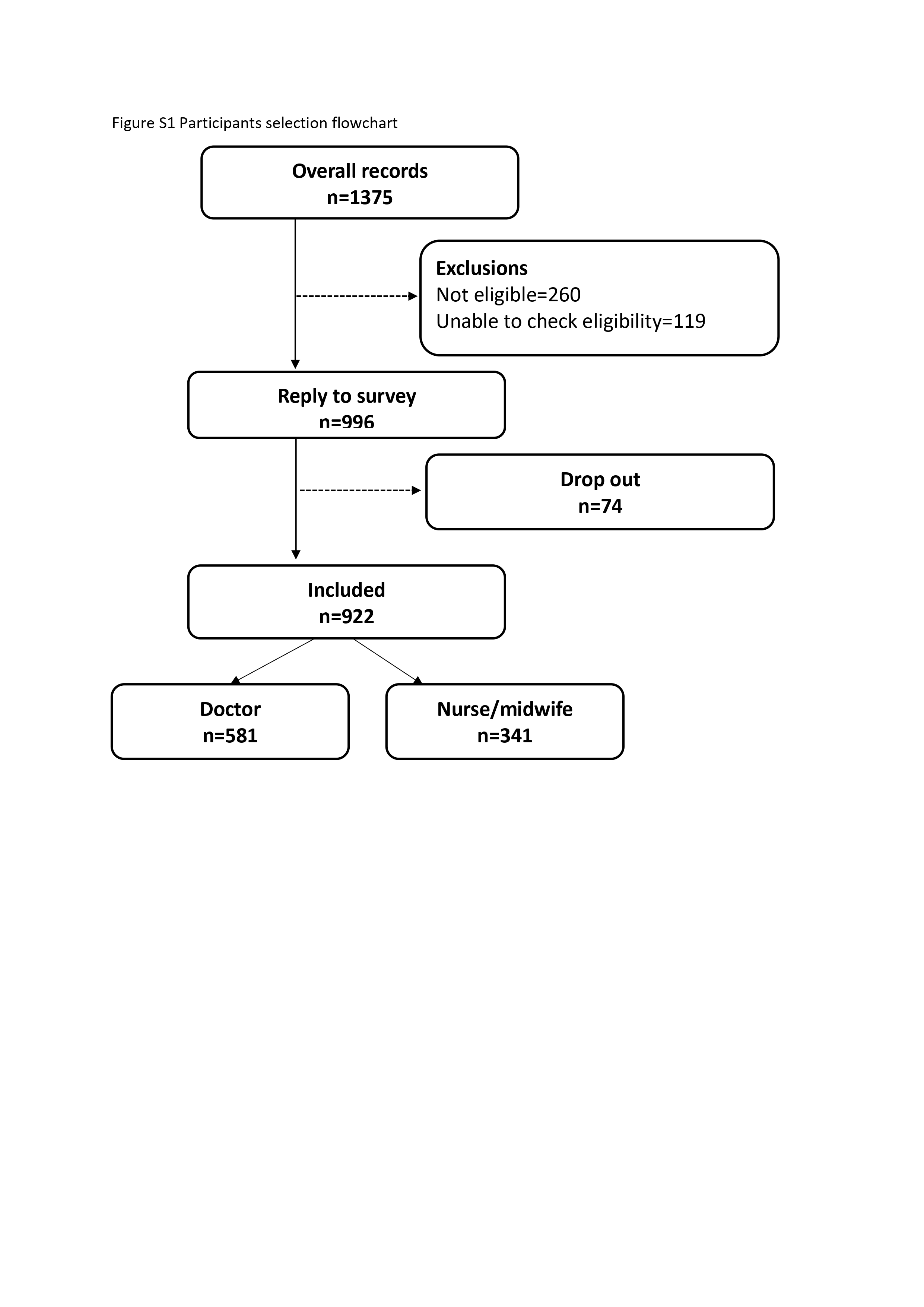

Supplement: Supplementary file 1 — Supplementary Material 1 [file 12992_2024_1095_MOESM1_ESM.jpg]
