# Supplementary material for: Awareness and knowledge regarding female genital schistosomiasis among European healthcare workers: a cross-sectional online survey
Source: Global Health. 2025 Jan 8;21:2. doi: 10.1186/s12992-024-01095-z (PMC11715917; doi:10.1186/s12992-024-01095-z)
Supplement: Supplementary file 2 — Supplementary Material 2 [file 12992_2024_1095_MOESM2_ESM.docx]

Supplementary material

Table S1. Estimates of the prevalence of FGS awareness and 95% confidence intervals and crude and adjusted prevalence ratios (PR) of FGS awareness among medical practitioners in European countries

|  | n/N | | p (CI95%) | | | Crude PR | Adjusted PR |
| --- | --- | --- | --- | --- | --- | --- | --- |
| **Total** | 254/ 581 | | 43.7(39.6, 47.9) | | |  |  |
| **Gender** |  | |  | | |  |  |
| Women | 123/ 317 | | 38.8(33.4, 44.4) | | | ref | ref |
| Men | 129/ 258 | | 50.0(43.7, 56.3) | | | 1.29 (1.07, 1.55) | 1.22 (1.02, 1.45) |
| **Age group** |  | |  | | |  |  |
| <34 | 71/176 | | 40.3(33.0, 48.0) | | | ref | ref |
| 35-44 | 65/150 | | 43.3(35.3, 51.7) | | | 1.07 (0.83, 1.39) | 1.04 (0.82, 1.31) |
| 45-54 | 56/117 | | 47.9(38.5, 57.3) | | | 1.19 (0.91, 1.54) | 1.15 (0.9, 1.47) |
| 55+ | 62/138 | | 44.9(36.5, 53.6) | | | 1.11 (0.86, 1.44) | 1.04 (0.81, 1.33) |
| **Country of practice** |  | |  | | |  |  |
| Germany | 74/176 | | 42.1(34.7, 49.7) | | | ref | ref |
| Italy | 67/139 | | 48.2(39.7, 56.8) | | | 1.15 (0.9, 1.46) | 1.04 (0.82, 1.31) |
| UK, Portugal, France | 36/166 | | 36.0(26.6, 46.2) | | | 0.86 (0.63, 1.17) | 0.89 (0.67, 1.17) |
| Other | 77/100 | | 46.4(38.6, 54.3) | | | 1.1 (0.87, 1.4) | 1.00 (0.80, 1.25) |
| **Years of experience** |  | |  | | |  |  |
| 0 to 9 | 78/203 | | 38.4(31.7, 45.5) | | | ref |  |
| 10 to 29 | 112/239 | | 46.9(40.4, 53.4) | | | 1.22 (0.98, 1.52) | na |
| 30+ | 51/110 | | 46.4(36.8, 56.1) | | | 1.21 (0.92, 1.57) | na |
| **Work in the field of STI** | |  | |  |  | |  |
| No | | 208/ 516 | | 40.3(36.1, 44.7) | ref | | ref |
| Yes | | 46/65 | | 70.8(58.2, 81.4) | 1.76 (1.45, 2.12) | | 1.2 (0.99, 1.45) |
| **Work with travellers/**  **migrant populations** | |  | |  |  | |  |
| No | | 164/456 | | 36.0(31.6, 40.6) | ref | | ref |
| Yes | | 90/125 | | 72.0(63.2, 79.7) | 2 (1.7, 2.36) | | 1.33 (1.1, 1.59) |
| **Workplace** | |  | |  |  | |  |
| Research/University hospital | | 121/249 | | 48.6(42.2, 55.0) | ref | |  |
| Non-university hospital | | 74/132 | | 56.1(47.2, 64.7) | 1.15 (0.95, 1.41) | | na |
| Outpatient/medical practice | | 50/176 | | 28.4(21.9, 35.7) | 0.58 (0.45, 0.76) | | na |
| Other | | 7/20 | | 35.0(15.4, 59.2) | 0.72 (0.39, 1.33) | | na |
| **Specialization** | |  | |  |  | |  |
| None | | 9/28 | | 32.1(15.9, 52.4) | 0.47 (0.27, 0.81) | | 0.52 (0.28, 0.95) |
| Occupational medicine | | 5/30 | | 16.7(5.6, 34.7) | 0.24 (0.11, 0.54) | | 0.28 (0.12, 0.64) |
| Urology | | 11/31 | | 35.5(19.2, 54.6) | 0.52 (0.32, 0.84) | | 0.61 (0.37, 1.00) |
| Gynaecology | | 36/91 | | 39.6(29.5, 50.4) | 0.57 (0.44, 0.75) | | 0.67 (0.51, 0.88) |
| Internal medicine | | 17/67 | | 25.4(15.5, 37.5) | 0.37 (0.24, 0.56) | | 0.42 (0.27, 0.65) |
| Infectiology/travel/tropical medicine | | 146/212 | | 68.9(62.2, 75.0) | ref | | ref |
| Family/General medicine | | 30/122 | | 24.6(17.3, 33.2) | 0.36 (0.26, 0.49) | | 0.42 (0.3, 0.59) |

Note: Years of experience, and workplace were not included in multiple Poisson regression model due to multicollinearity.

Table S2. Source of information about FGS among medical practitioners aware of FGS in European countries

|  | n/N | % |
| --- | --- | --- |
| Doctors (n=254) |  |  |
| Academic curriculum | 88/254 | 34.7 |
| Conference | 65/254 | 25.6 |
| Workshop | 59/254 | 23.2 |
| Scientific literature | 72/254 | 28.4 |
| Personal experience | 37/254 | 14.6 |
| Patient contact | 45/254 | 17.7 |
| Nurses/midwives (n=41) |  |  |
| Academic curriculum | 15/41 | 36.6 |
| Conference | 5/41 | 12.2 |
| Workshop | 7/41 | 17.1 |
| Scientific literature | 7/41 | 17.1 |
| Personal experience | 4/41 | 9.8 |
| Patient contact | 1/41 | 2.4 |

Table S3 Estimates of the prevalence of different levels of FGS knowledge among European medical practitioners

|  | No knowledge | | Low knowledge | | Medium knowledge | |
| --- | --- | --- | --- | --- | --- | --- |
|  | **n** | p(CI95%) | **n** | p(CI95%) | **n** | p(CI95%) |
| **Gender** |  |  |  |  |  |  |
| Men | 159/258 | 61.6(55.4, 67.6) | 76/258 | 29.5(24.0, 35.4) | 23/258 | 8.9(5.7, 13.1) |
| Women | 229/317 | 72.2(67.0, 77.1) | 70/317 | 22.1(17.6, 27.1) | 18/317 | 5.7(3.4, 8.8) |
| **Age group** |  |  |  |  |  |  |
| <34 | 122/176 | 69.3(61.9, 76.0) | 41/176 | 23.3(17.3, 30.3) | 13/176 | 7.4(4, 12.3) |
| 35-44 | 101/150 | 67.3(59.2, 74.8) | 35/150 | 23.3(16.8, 30.9) | 14/150 | 9.3(5.2, 15.2) |
| 45-54 | 75/117 | 64.1(54.7, 72.8) | 38/117 | 32.5(24.1, 41.8) | 4/117 | 3.4(0.9, 8.5) |
| 55+ | 95/138 | 68.8(60.4, 76.5) | 33/138 | 23.9(17.1, 31.9) | 10/138 | 7.3(3.5, 12.9) |
| **Country of practice** |  |  |  |  |  |  |
| Germany | 122/176 | 69.3(61.9, 76.0) | 41/176 | 23.3(17.3, 30.3) | 13/176 | 7.4(4, 12.3) |
| Italy | 88/139 | 63.3(54.7, 71.3) | 38/139 | 27.3(20.1, 35.5) | 13/139 | 9.4(5.1, 15.5) |
| UK+PT+France | 73/100 | 73.0(63.2, 81.4) | 21/100 | 21.0(13.5, 30.3) | 6/100 | 6(2.2, 12.6) |
| Other | 110/166 | 66.3(58.5, 73.4) | 47/166 | 28.3(21.6, 35.8) | 9/166 | 5.4(2.5, 10) |
| **Years of experience** |  |  |  |  |  |  |
| 0 to 9 | 143/203 | 70.4(63.7, 76.6) | 45/203 | 22.2(16.7, 28.5) | 15/203 | 7.4(4.2, 11.9) |
| 10 to 29 | 157/239 | 65.7(59.3, 71.7) | 67/239 | 28.0(22.4, 34.2) | 15/239 | 6.3(3.6, 10.1) |
| 30+ | 73/110 | 66.4(56.7, 75.1) | 28/110 | 25.5(17.6, 34.7) | 9/110 | 8.2(3.8, 15) |
| **Workplace** |  |  |  |  |  |  |
| Research/University hospital | 160/249 | 64.3(58.0, 70.2) | 71/249 | 28.5(23.0, 34.6) | 18/249 | 7.2(4.3, 11.2) |
| Non-university hospital | 70/132 | 53.0(44.2, 61.8) | 47/132 | 35.6(27.5, 44.4) | 15/132 | 11.4(6.5, 18.1) |
| Outpatient/medical practice | 143/176 | 81.3(74.7, 86.7) | 25/176 | 14.2(9.4, 20.3) | 8/176 | 4.6(2, 8.8) |
| Other | 16/20 | 80.0(56.3, 94.3) | 4/20 | 20.0(5.7, 43.7) | 0/20 | 0(0, 0.2) |
| **Specialization** |  |  |  |  |  |  |
| Infectiology/travel/tropical | 94/212 | 44.3(37.5, 51.3) | 88/212 | 41.5(34.8, 48.5) | 30/212 | 14.2(9.8, 19.6) |
| None | 24/28 | 85.7(67.3, 96.0) | 2/28 | 7.1(0.9, 23.5) | 2/28 | 7.1(0.9, 23.5) |
| Occupational | 26/30 | 86.7(69.3, 96.2) | 3/30 | 10.0(2.1, 26.5) | 1/30 | 3.3(0.1, 17.2) |
| Urology | 25/31 | 80.7(62.5, 92.6) | 5/31 | 16.1(5.5, 33.7) | 1/31 | 3.2(0.1, 16.7) |
| Gynaecology | 67/91 | 73.6(63.4, 82.3) | 21/91 | 23.1(14.9, 33.1) | 3/91 | 3.3(0.7, 9.3) |
| Internal medicine | 56/67 | 83.6(72.5, 91.5) | 10/67 | 14.9(7.4, 25.7) | 1/67 | 1.5(0, 8.0) |
| Family/General | 101/122 | 82.8(74.9, 89.0) | 18/122 | 14.8(9.0, 22.3) | 3/122 | 2.5(0.5, 7.0) |
| **Work with travellers/migrant populations** |  |  |  |  |  |  |
| No | 343/456 | 75.2(71.0, 79.1) | 96/456 | 21.1(17.4, 25.1) | 17/456 | 3.7(2.2, 5.9) |
| Yes | 50/125 | 40.0(31.3, 49.1) | 51/125 | 40.8(32.1, 50.0) | 24/125 | 19.2(12.7, 27.2) |
| **Work in the field of STI** |  |  |  |  |  |  |
| No | 361/516 | 70.0(65.8, 73.9) | 123/516 | 23.8(20.2, 27.8) | 32/516 | 6.2(4.3, 8.6) |
| Yes | 32/65 | 49.2(36.6, 61.9) | 24/65 | 36.9(25.3, 49.8) | 9/65 | 13.9(6.5, 24.7) |
| **Work endemic country** |  |  |  |  |  |  |
| No | 224/308 | 72.7(67.4, 77.6) | 63/308 | 20.5(16.1, 25.4) | 21/308 | 6.8(4.3, 10.2) |
| Yes | 147/251 | 58.6(52.2, 64.7) | 84/251 | 33.5(27.7, 39.7) | 20/251 | 8(4.9, 12.0) |

Table S4 Estimates of the crude prevalence ratios of levels of FGS knowledge among European medical practitioners

|  | **Low knowledge vs. No knowledge** | **Medium vs. No knowledge** |
| --- | --- | --- |
| **Gender** |  |  |
| Female |  |  |
| Male | 1.38 (1.05,1.82) | 1.73 (0.96,3.12) |
| **Age group** |  |  |
| <34 |  |  |
| 35-44 | 1.02 (0.69,1.51) | 1.26 (0.62,2.58) |
| 45-54 | 1.34 (0.92,1.94) | 0.53 (0.18,1.56) |
| 55+ | 1.02 (0.69,1.52) | 0.99 (0.45,2.17) |
| **Country of practice** |  |  |
| Germany |  |  |
| Italy | 1.20 (0.82,1.75) | 1.34 (0.65,2.76) |
| UK, Portugal, France | 0.89 (0.56,1.41) | 0.79 (0.31,1.99) |
| Other | 1.19 (0.83,1.7) | 0.79 (0.35,1.77) |
| **Years of experience** |  |  |
| 0 to 9 |  |  |
| 10 to 29 | 1.25 (0.9,1.73) | 0.92 (0.46,1.82) |
| 30+ | 1.16 (0.77,1.74) | 1.16 (0.53,2.53) |
| **Workplace** |  |  |
| Research/University hospital |  |  |
| Non-university hospital | 1.31 (0.97,1.75) | na |
| Outpatient/medical practice | 0.48 (0.32,0.73) | na |
| Other | 0.65 (0.26,1.6) | na |
| **Specialization** |  |  |
| Infectiology/travel/tropical |  |  |
| None | 0.16 (0.04,0.61) | 0.32 (0.08,1.25) |
| Occupational | 0.21 (0.07,0.63) | 0.15 (0.02,1.08) |
| Urology | 0.34 (0.15,0.78) | 0.16 (0.02,1.12) |
| Gynaecology | 0.49 (0.33,0.74) | 0.18 (0.06,0.56) |
| Internal medicine | 0.31 (0.17,0.57) | 0.07 (0.01,0.52) |
| Family/General | 0.31 (0.2,0.49) | 0.12 (0.04,0.38) |
| **Work with travellers/migrant populations** |  |  |
| No |  |  |
| Yes | 2.31 (1.78,3) | 6.87 (3.89,12.14) |
| **Work in the field of STI** |  |  |
| No |  |  |
| Yes | 1.69 (1.2,2.37) | 2.7 (1.38,5.25) |
| **Work endemic country** |  |  |
| No |  |  |
| Yes | 1.66 (1.26,2.19) | 1.4 (0.78,2.5) |
